# Transient Oscillations of Neural Firing Rate Associated With Routing of Evidence in a Perceptual Decision

**DOI:** 10.1523/JNEUROSCI.2200-22.2023

**Published:** 2023-09-13

**Authors:** Naomi N. Odean, Mehdi Sanayei, Michael N. Shadlen

**Affiliations:** ^1^Zuckerman Mind Brain Behavior Institute, Department of Neuroscience, Columbia University, New York, New York 10025; ^2^Howard Hughes Medical Institute, Columbia University, New York, New York 10025; ^3^Kavli Institute, New York, New York 10025

**Keywords:** beta oscillations, decision making, information routing, lateral intraparietal area, random dot motion

## Abstract

To form a perceptual decision, the brain must acquire samples of evidence from the environment and incorporate them in computations that mediate choice behavior. While much is known about the neural circuits that process sensory information and those that form decisions, less is known about the mechanisms that establish the functional linkage between them. We trained monkeys of both sexes to make difficult decisions about the net direction of visual motion under conditions that required trial-by-trial control of functional connectivity. In one condition, the motion appeared at different locations on different trials. In the other, two motion patches appeared, only one of which was informative. Neurons in the parietal cortex produced brief oscillations in their firing rate at the time routing was established: upon onset of the motion display when its location was unpredictable across trials, and upon onset of an attention cue that indicated in which of two locations an informative patch of dots would appear. The oscillation was absent when the stimulus location was fixed across trials. We interpret the oscillation as a manifestation of the mechanism that establishes the source and destination of flexibly routed information, but not the transmission of the information *per se*.

**Significance Statement** It has often been suggested that oscillations in neural activity might serve a role in routing information appropriately. We observe an oscillation in neural firing rate in the lateral intraparietal area consistent with such a role. The oscillations are transient. They coincide with the establishment of routing, but they do not appear to play a role in the transmission (or conveyance) of the routed information itself.

## Introduction

Human and animal behavior is remarkably flexible. We can execute a particular action in response to a wide variety of prompts. In a laboratory setting, a monkey might move its eyes to a location because a visual target had been flashed there a moment ago or because a visual stimulus at another location (or a tone) predicts a reward for this eye movement. In both scenarios, there is an elevation of the firing rate of neurons that direct attention and orienting responses to the target. In the first case, the sensory input prompting this activation comes from neurons in the visual cortex with receptive fields that overlap the target location. In the second, the sensory input is from visual cortical neurons with receptive fields that do not overlap the target (or from auditory cortex). In the setting of decision-making, we might say that there are many possible sources of evidence that could bear on the decision to choose a particular response. Therein lie the seeds of a routing problem that is central to cognition (Zylberberg et al., 2010). A general mechanism for routing is currently unknown, but the topic arises in the study of spatial attention and cognitive control, where oscillatory activity and/or synchronous spiking are thought to play a role (Saalmann et al., 2007; Pesaran et al., 2008; Dean et al., 2012; Gregoriou et al., 2012; Lee et al., 2013; Drebitz et al., 2018; Fiebelkorn et al., 2018, 2019; Grothe et al., 2018; Stanley et al., 2018).

We examined information routing in the context of perceptual decision-making, using a well-studied direction discrimination task (Newsome and Pare, 1988; Britten et al., 1992). The subject, a rhesus monkey, must determine the net direction of dynamic random dots, only a fraction of which are informative at any moment. The decision is indicated by a saccadic eye movement to one of two choice targets located on opposite sides of the random dot display. The decision is easy when many of the dots are moving coherently (strong motion); it is difficult when most of the dots are randomly replotted and only a small fraction of the dots are informative (weak motion). To perform well, the subject must accumulate noisy evidence over time. This accumulation is reflected in the activity of neurons in the lateral intraparietal area (LIP) with receptive fields that overlap one of the choice targets (Shadlen and Newsome, 1996), an observation that presupposes a solution to a routing problem. Momentary evidence from direction selective neurons in area MT, with receptive fields that overlap the motion, must be routed, directly or indirectly, to neurons in LIP that represent the choice targets (Salzman et al., 1992; Shadlen and Kandel, 2021). This routing could not be anticipated by evolution. In some cases, it might be established through learning, while in others it may need to be established on the fly. Here, we focus on the latter scenario.

We used two tasks that require a solution to the routing problem on each experimental trial. In the first, a visual cue instructs the monkey to make its decision about one of two patches of random dots (cued attention task). In the second, a single patch of motion appears at an unpredictable location (variable location task). In both tasks, LIP neurons exhibit decision-related activity during motion viewing, consistent with successful routing on most trials. We reasoned that the routing must be established after the onset of the attention cue or the motion stimulus and before the neurons in LIP begin to represent the accumulating evidence. We observed a prominent oscillation in the firing rates of single neurons in these epochs. The oscillation is aligned to the onset of the instructive cue in the cued attention task and to the onset of the motion stimulus itself in both tasks. The oscillations are brief and limited to the epoch preceding the representation of the accumulating evidence. We propose that they are signatures of the mechanisms that establish the routing of evidence to the site of its incorporation in a decision, but they do not appear to play a role during the information transfer accompanying decision formation.

## Materials and Methods

Four adult male rhesus monkeys (*Macaca mulatta*) were implanted with a titanium headpost (Rogue Research) and a plastic (Peek) recording chamber (Crist Instruments). Previously published data from two adult female rhesus monkeys were also analyzed. The placement of the chamber was guided by 3D reconstruction of MRI scans (OsiriX DICOM Viewer, Pixmeo) to ensure access to area LIP along the left intraparietal sulcus. In the experiments, the monkeys were seated in a primate chair (Crist Instruments) that was custom fit to support the monkey’s size and weight during head stabilization, allowing the monkey to adjust its posture below the head and thus prevent potential discomfort associated with head stabilization. Extracellular single-neuron recordings were made using quartz-coated tungsten microelectrodes (Thomas Recording) or 16-channel V-probes or S-probes (Plexon), which were advanced (Mini Matrix drive, Thomas Recording) through a metal guide tube seated in a plastic grid. Electrical recordings were filtered and amplified (Ominplex recording system, Plexon). Waveforms identified as single neuron action potentials were saved, and each occurrence was assigned a spike time. The quality of isolation was confirmed offline based on interspike interval and clustering based on principal component analysis of the waveforms (Plexon Offline Sorter).

### Experimental design and statistical analyses

All procedures were approved by the Columbia University Institutional Animal Care and Use Committee and conform to the National Institutes of Health’s *Guide for the care and use of laboratory animals* (National Research Council, 2011).

### Behavioral tasks

Monkeys were trained to perform a variety of oculomotor and perceptual tasks that required the monkey to maintain the gaze on a fixation point (FP) and to make saccadic eye movements to visual targets in the periphery (see Table 2). Eye position (gaze angle) was measured with high speed video tracking (EyeLink 1000, SR research). The acceptance window for eye position during fixation was a square ± 1.5° from the FP (i.e., 9 deg^2^). Here and throughout, °, or deg, stands for degrees visual angle. For saccades to peripheral targets, the acceptance window was a ±5° square around the target center. The criteria were relaxed for eccentricities exceeding 12°.

#### Cued attention task

**Figure 1.**
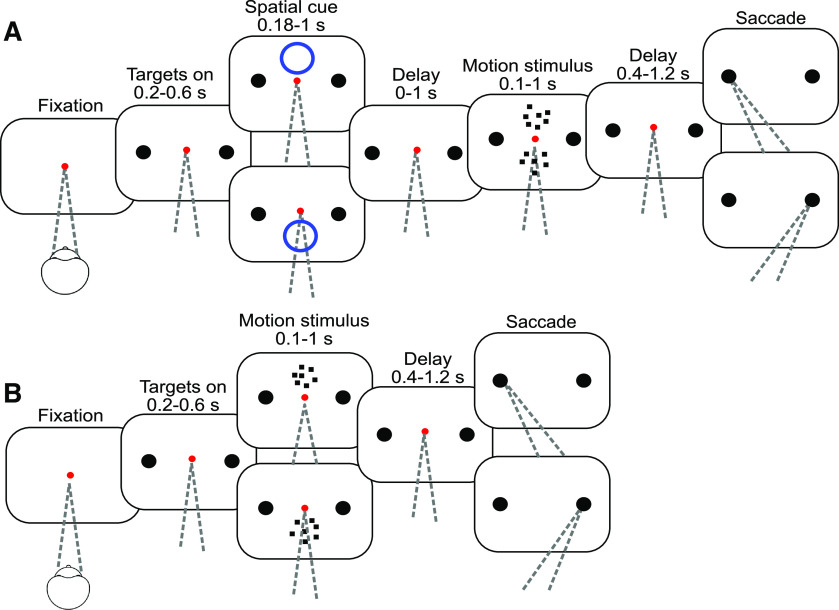
Task flow. ***A***, Cued attention task. After the monkey acquires fixation, two choice targets appear, followed by a brief spatial cue (blue circle). One choice target is positioned in the response field of the recorded neuron, and the other at the same elevation and azimuth in the opposite hemifield. After a delay, two random dot motion patches appear. The motion strength is the same in the two patches, but the directions may be the same or opposite. When the FP is extinguished, the monkey indicates the direction of the cued motion with a saccade to the left or right choice target and receives a reward if the choice corresponds to the direction of the cued patch. ***B***, Variable location task. Same as in ***A***, except that there is no attention cue, and only one patch of motion is shown, either above or below the point of fixation. For two monkeys, the motion direction was not restricted to the horizontal axis. For these monkeys, one target was positioned in the response field and the other was positioned at the same distance from the FP, so that a line passing through the two targets and the FP parallels the direction of motion.

Two monkeys were trained to perform a variation of a random dot motion direction-discrimination task used in previous studies (e.g., see Roitman and Shadlen, 2002), in which two motion patches are shown, but only one is informative. In this cued attention task (Fig. 1), the monkey initiates a trial by fixating on a central red dot against a black background. After 0.35 s two white targets appear, each with a diameter of 0.5 deg (1.5 cd/m^2^). One target is positioned at the center of the response field of the recorded neuron, and the other at the same elevation and eccentricity in the opposite hemifield. Target onset is followed by a delay period, drawn from a truncated exponential distribution as follows:

$$f(t)=\{\begin{array}{ll} \frac{\alpha }{\tau }e^{-\frac{t-t_{\text{min}}}{\tau }} & t_{\text{min}}\leq t\leq t_{\text{max}}\\ 0 & \text{otherwise} \end{array}$$

where τ = 0.1, *t*_min_ = 0.2 s, *t*_max_ = 0.6 s, and *α* is chosen to ensure the total probability is unity. The expectation of *t* is less than *t*_min_ + τ, owing to truncation. In what follows, all variable delay periods are described by a range, *t*_min_ to *t*_max_, and *τ* in Equation 1.

The cue, a blue ring with a diameter of 5° centered 4.5° directly above or below the FP, is flashed on the screen for 0.18-1 s (*τ* = 0.2 s). After a 0-1 s delay (*τ* = 0.15 s), two motion patches appear. The motion stimulus consists of white dots (1.5 cd/m^2^) on a black background. New sets of dots are shown for the first three frames. The fourth frame replots the dots from frame one such that with probability *p* a dot is displaced by Δ*x* or, with probability 1 – *p*, replaced at a random location. We refer to *p* as the motion strength or coherence (coh) and indicate the direction of motion by sign. The sequence continues such that every frame *n* updates the dots from frame *n* – 3. The monkey is encouraged to attend to the cued motion patch while ignoring the irrelevant motion in the uncued stimulus. The direction of motion of the uncued stimulus is the same as that of the cued stimulus on one-third of trials and opposite on two-thirds. We find this ratio works to minimize the influence of the uncued motion stimulus on the choices. In this task, motion is always leftward or rightward. For each trial, the strength of the net motion is drawn with equal probability from the set (0, 0.032, 0.064, 0.128, 0.256, 0.512). We refer to these values as % coherence and indicate the direction with ±. The strength of motion for the two patches is matched to prevent the monkey from responding based on the easier motion stimulus rather than the relevant one. The cued location is assigned with equal probability to one of the two locations for each trial. Motion lasts for 0.1-1.5 s (*τ* = 0.35 s), followed by a 0.4-1.2 s delay (*τ* = 0.15 s). After this delay, the red FP disappears and the monkey indicates its response by a saccade to a left or right choice target.

#### Variable location task

In the variable location task, only one motion patch is shown, and there is no attention cue. For Monkeys Ap and Dz, motion is not restricted to the horizontal axis. The choice targets are positioned along the direction of motion, symmetrically on an imaginary line parallel to the motion direction and through the center of the random dot motion display. One target is centered in the neural response field (location *A*); and the other is at location *B*, such that a virtual line *AB* passed through the FP (*F*), and AF≅BF). The opposing directions of motion parallel *AB*. Monkey Dm was only trained on horizontal motion, so the two targets share the same elevation. The task is otherwise similar to the cued attention task.

The random dot motion is confined to two circular apertures (5° diameter) centered at the same elevation above and/or below the FP. The locations are determined by establishing the extent of the neural response field so as to avoid overlap. The monkey performs a series of delayed saccades (see Mapping tasks), and we ensure that saccadic targets (white spots, 0.5° diameter) do not elicit a visual or memory response when they appear at the locations where the random dot motion and attention cues are to be displayed.

For Monkeys Ap and Dm, motion is displayed for 0.1-1 s (Eq. 1, *τ* = 0.25 s) and is followed by a 0.4-1.2 s delay (*τ* = 0.15 s). After this delay, the FP disappears and the monkey indicates its decision by making a saccade to one of the choice targets. Motion strengths are drawn from the same distribution as in the cued attention task for Monkeys Dm and Ap; the strongest coherence is not included for Monkey Dz. For this monkey, we used a free response (choice-response time) design. The task is identical to the controlled duration version, except that a saccadic response is accepted any time after motion onset. For all monkeys, correct choices are rewarded with a drop of juice. Trials with 0% coherent motion are rewarded with a probability of 50% regardless of the monkey’s choice. Experiments were conducted in alternating blocks of 120 trials with either a fixed or variable stimulus location. In a fixed location block, the motion stimulus appeared in one location for 60 consecutive trials and then appeared in the other location for 60 trials.

Monkeys were seated facing the video display (viewing distance, 57 cm). In the cued attention task, and for Monkeys Ap and Dz in the variable location task, stimuli were shown on a 40 cm CRT monitor with a 75 Hz frame rate (NEC, MultiSync FP1370). For Monkey Dm in the variable location task, stimuli were shown on a 54 cm LCD with an effective refresh rate of 60 frames per second (Acer, HN274H). For this display, the interval between updated frames was reduced from every third frame to every second frame. The dot displacement was adjusted to achieve consistent speeds across the two display types (typically 5°/s).

#### Mapping tasks

We conducted two mapping tasks to screen neurons for study. In both, the monkey maintains its gaze on a central FP, and initiates a saccade when the FP is extinguished. In the memory saccade task, after attaining central fixation and a random delay (0.2-1 s, *τ* = 0.2), a white target (0.5° diameter) is flashed in the periphery (0.2 s). After a memory delay (0.7-1.2 s, *τ* = 0.15) from target onset, the FP is extinguished and the monkey is free to saccade to the cued location to receive a juice reward. The overlap saccade task is identical to the memory saccade, except the target remains visible throughout the delay period and the saccade. We refer to both of these tasks as oculomotor delayed response (ODR tasks) (Hikosaka and Wurtz, 1983; Funahashi et al., 1989).

### Neuron selection and recording

Recording sites were selected by 3D reconstruction of anatomic MRI scans. The electrode was advanced along the intraparietal sulcus at positions that are thought to correspond to the ventral portion of the lateral intraparietal area (Lewis and Van Essen, 2000) where one encounters many neurons with visual and perisaccadic responses. Within the putative ventral portion of the lateral intraparietal area, we mapped all well-isolated units using the ODR tasks. A neuron was included in the dataset if it showed spatially selective persistent activity during the delay period of memory saccades and if the neural response field allowed for a task geometry compatible with the monkey’s training. We excluded neurons *post hoc* if we obtained <240 trials before the signal-to-noise deteriorated to the point that the spike waveform was not adequately isolated (7 of 71 neurons in the variable location task).

In the cued attention task, a 16 channel probe was used to record several neurons simultaneously. All channels were screened with the memory-guided saccade task. The recording probe was positioned to maximize the number of recorded units showing memory activity. The task objects could not be placed optimally for all cells, but nearby cells tended to have similar response fields. The task geometry was optimized for the best isolated channel. This yielded 1-7 simultaneously recorded cells with acceptable task geometry: a choice target roughly centered in the response field and both motion patches outside the response field. Cells were sorted offline as for single electrodes. Particular attention was paid to whether waveform principal components or spike rate changed over time to ensure that the same cell was recorded throughout the session. Monkeys performed an ODR trial to each target location after every 40 trials on the motion task, and thorough screening was repeated at the end of the session to ensure that response fields were constant throughout the session. If a cell showed a change in any of these parameters, trials after that change were excluded from analysis. Occasionally, a new waveform appeared during the recording session. It was included in the analysis if it (1) was well isolated from background noise, (2) exhibited consistent waveform, principal components, spike rate, and response preference in the interleaved ODR trials, and (3) showed an appropriate response field in the postsession screening tasks. Adjacent neurons from the first seven sessions were checked for cospiking. As no duplicate neurons were identified in these sessions, this check was not continued.

### Data analysis

**Figure 3.**
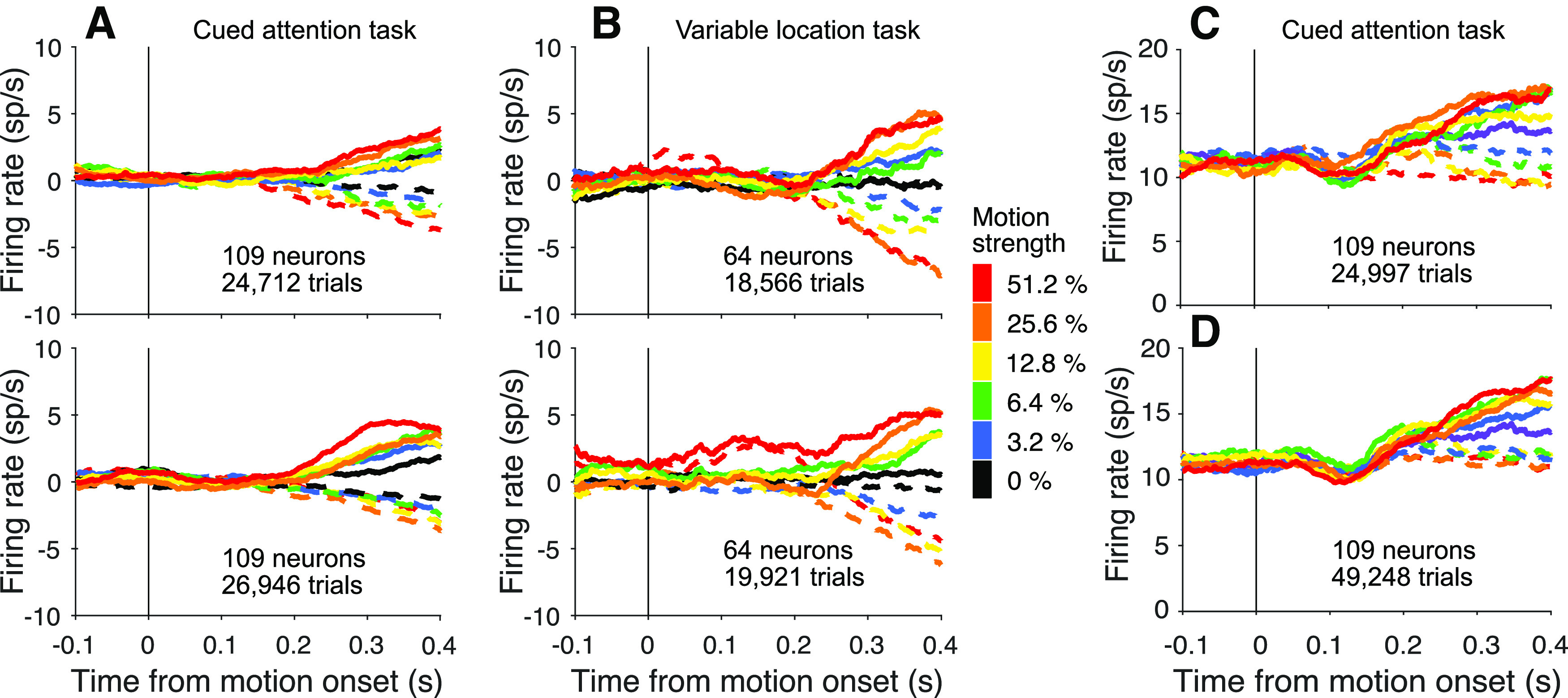
LIP neurons reflect evidence from the attended patch of motion. ***A***, Activity of 109 neurons studied in the cued attention task when the upper patch (top) and lower patch (bottom) were cued as informative (combined data from Monkeys Dm and Np). Responses are detrended by neuron, via subtraction of the mean firing rate, as function of time, on the lowest coherences (0% and ±3.2%). Errors on non-zero coherences are excluded. The neurons reflect the formation of decision from information derived from the upper and lower visual field. ***B***, Activity of 36 neurons studied in the variable location task when the motion patch appeared in the upper (top) or lower (bottom) location. Combined activity of neurons from Monkeys Ap and Dz only, as neurons from Monkey Dm recorded in this task did not show dependence on the strength of motion. We report statistics from this task both with and without neurons from Monkey Dm. ***C***, ***D***, Comparison of responses in the cued attention task when motion patches had the same (***C***) or opposite (***D***) directions. The graphs use the combined data from both monkeys without detrending.

Peristimulus time histograms were generated by aligning spike times to an event of interest and finding the average number of spikes, across trials, in time bins relative to the event. Time bins are 5 ms for averages across neurons and 10 ms for single neurons. For the firing rate versus time graphs in Figure 3, the rates were obtained by convolving the point process, δ(*t* – *s_i_*), where *s_i_* are spike times, with a noncausal boxcar filter of width 100 ms. This smoothing is not applied to any other plot or analysis, as it obscures the oscillations of interest. To better visualize the decision-related activity, the responses in Figure 3 are detrended. For each neuron, the average response to the 0 and ± 3.2% coherences is subtracted. Figures show the average across neurons, with each neuron weighted by the number of recorded trials. Across the two tasks, 8 of 173 neurons show a preference for the ipsilateral direction during the motion viewing epoch. For these neurons, the sign of the motion is reversed in analyses of signed coherence (see Fig. 3).

#### Behavior

The decision process leading to leftward and rightward choices is affected by the direction and strength of motion as well as the duration of the stimulus. The durations were controlled by the experimenter for the three monkeys displayed in Figure 2. For the fourth monkey, Dz, we used a free response (choice-response time) design. Decision formation in both designs is explained by a process of bounded accumulation of noisy evidence, also known as bounded drift-diffusion (Kiani et al., 2008). Momentary motion evidence is integrated over time until it reaches one of two bounds (± *B*) or the evidence stream is turned off. The influence of the motion evidence depends on the signed motion strength (*C*) and on a drift rate parameter (*κ*).

$$dV=\kappa (C+C_{0})dt+dW,$$

where *W* is a standard Wiener process (i.e., *dW* is a sample drawn from a Normal distribution, $N\{0,\sqrt{dt}\})$. The initial state is $V_{t=0}=0$ and the process continues until |V(t)|≥B. The time of this termination governs the response time in a free response task (e.g., Monkey Dz), and simply curtails further integration when the stimulus duration is controlled experimentally. If the decision process is terminated when integrated evidence reaches a bound, the chosen direction is the sign of the bound reached. If a bound has not yet been reached before the evidence stream is turned off (at $t=t_{\text{dur}}$), the chosen direction depends on the sign of the unabsorbed integrated evidence. The choice probability was modeled by fitting *B*, *κ*, and a bias term *C*_0_ expressed as an offset in signed motion strength (Hanks et al., 2011; Urai et al., 2019). These quantities are obtained by numerical solution of the Fokker-Planck equation, which yields a probability density comprising three components: (1) $f_{+}(t|t\leq t_{\text{dur}})$, the upper bound absorption times, (2) $f_{-}\left(t|t\leq t_{\text{dur}}\right)$, the lower bound absorption times, and (3) $f_{\text{un}}(V|t=t_{\text{dur}})$ the values of the unabsorbed *V* at $t=t_{\text{dur}}$, such that

$$\int_{0}^{t_{\text{dur}}}f_{+}(t)+f_{-}(t)dt+\int_{-B}^{+B}f_{\text{un}}(V_{t_{\text{dur}}})dV=1$$

**Figure:**
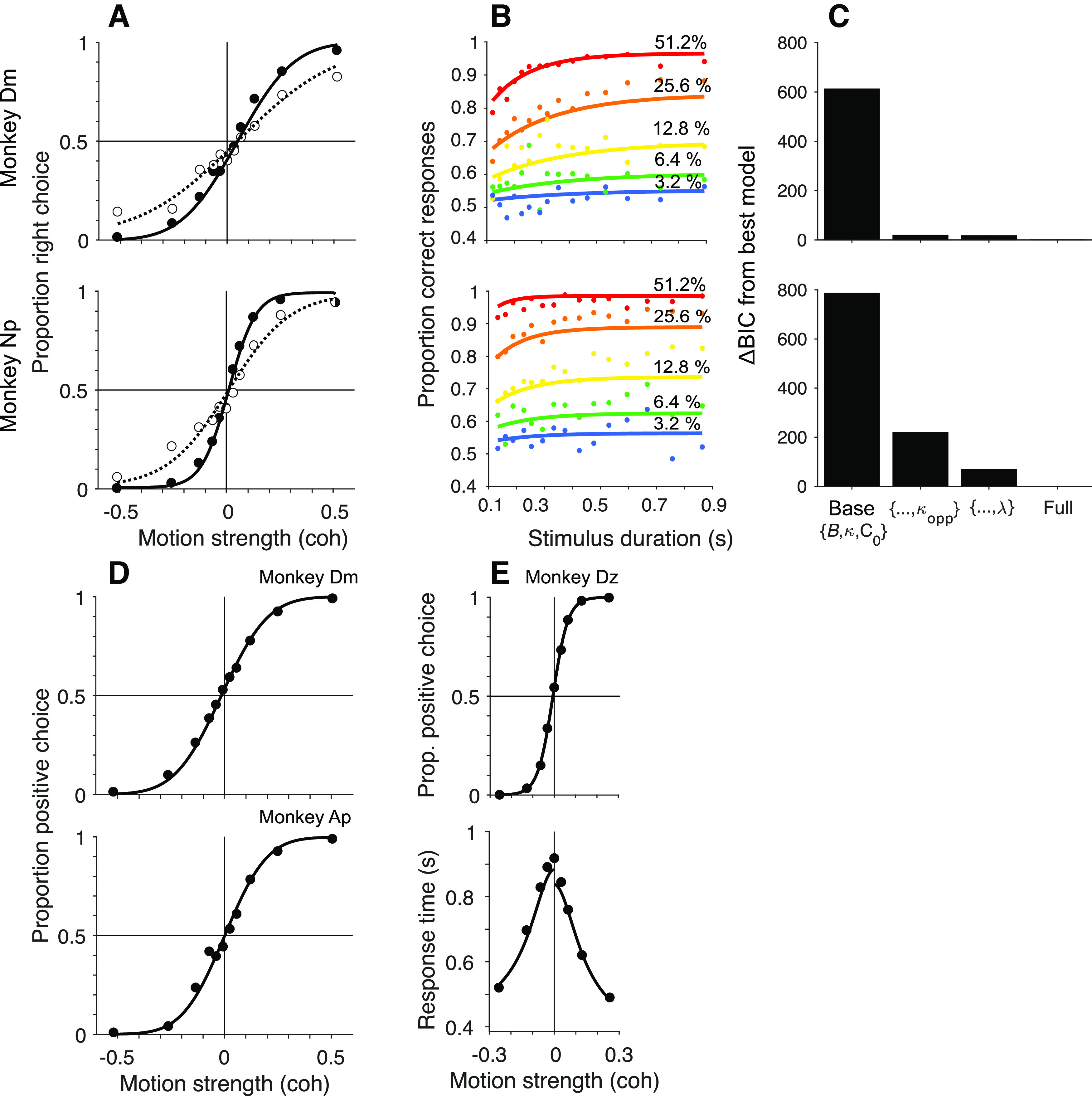
Behavior. ***A***, Cued attention task. Proportion of rightward choices is plotted as a function of signed motion strength of the cued patch (positive coherence signifies rightward). Filled and open symbols represent trials where the direction of the uncued motion patch was the same or opposite to the direction of the cued patch, respectively, combining trials for all viewing durations. Solid and dashed curves are fits of a bounded drift-diffusion model that incorporates misrouting owing to incomplete suppression of the uncued patch or attending to it erroneously on a fraction of trials. Curves indicate the expectation of the choice-proportions for the mean stimulus duration (top, Monkey Dm; bottom, Monkey Np). ***B***, Choice-accuracy improved as function of stimulus viewing duration in the cued attention task. Same data as in ***A***, grouped by motion strength (colors) and quantiles of stimulus duration. Curves are fits of the same model as in ***A***. ***C***, Comparison of fits to models of Equations 2, 4, and 5. Model comparison favors inclusion of $\kappa_{\text{opp}}$ and *λ* for both monkeys. ***D***, Variable location task with random stimulus durations. There is only one patch of motion. The proportion of choices in the positive direction (favoring the target in the neural response field) is plotted as as a function of signed coherence. The smooth curve is a fit to a simpler bounded drift-diffusion model. As in ***A***, the proportions reflect all stimulus durations, and the fit shows predictions for the mean duration (top, Monkey Dm; bottom, Monkey Ap). ***E***, Choice-response time version of the variable location task. The choices (top) and response times (bottom) are fit by a bounded drift-diffusion model. Fit parameters for all monkeys and conditions are in Table 1.

**Table 1:** Parameters for model fits^*a*^

Task	Monkey	*κ*	*B*	*C*_0_	$\kappa_{\text{opp}}$	*λ*
Cued attention	Dm	7.9	0.74	–0.047	4.6	0.029
Cued attention	Np	17.9	0.44	–0.011	7.9	0.006
Variable location	Dm	10.3	0.64	0.013	—	—
Variable location	Ap	11.4	0.61	–0.001	—	—
Variable location (FR)	Dz	17.1	0.74	–0.02	—	—

*^a^*Variables are defined in Equations 2, 4, and 5. Free response task (FR) includes two additional parameters: the expectations of the nondecision times, $T_{\text{nd}}^{\text{right}}=0.33$ and $T_{\text{nd}}^{\text{left}}=0.34$, which accounts for sensory and motor latencies that add to the decision time to explain the total response time.

The probability of a positive choice is as follows:

$${\tilde{P}}_{+}(C,t_{\text{dur}})=\int_{0}^{t_{\text{dur}}}f_{+}(t)dt+\int_{0}^{+B}f_{\text{un}}(V_{t_{\text{dur}}})dV_{t_{\text{dur}}}$$

and

$${\tilde{P}}_{-}(C,t_{\text{dur}})=1-{\tilde{P}}_{+}(C,t_{\text{dur}})$$

This specifies the base diffusion model without misrouting. The alternative models consist of (1) attention to the wrong patch and (2) incomplete suppression of the uncued motion patch. For the base model, the observed proportion of positive choices is $P_{+}(C,t_{\text{dur}})={\tilde{P}}_{+}(C,t_{\text{dur}})$. If the monkey attends to the wrong motion patch on a fraction of trials, *λ*, then


$$P_{+}(C,t_{\text{dur}})=\{\begin{array}{ll} {\tilde{P}}_{+}(C,t_{\text{dur}}) & \text{same direction}\\ \left(1-\lambda \right){\tilde{P}}_{+}(C,t_{\text{dur}})+\lambda {\tilde{P}}_{-}(C,t_{\text{dur}}) & \text{opposite direction} \end{array}$$

To model incomplete suppression of the uncued patch, we allow for a different value of *κ* in Equation 2 when the patches have the same or opposite directions

$$dV=\{\begin{array}{ll} \kappa (C+C_{0})dt+dW & \text{same direction}\\ \kappa_{\text{opp}}(C+C_{0})dt+dW & \text{opposite direction} \end{array}$$

where *C* is the signed coherence of the cued patch.

The model was fit separately for the two monkeys using maximum likelihood. The fitted parameters are (κ, *C*_0_, *B*) for the basic model without misrouting (df = 3). Erroneous routing and incomplete suppression add one degree of freedom apiece. We report the absolute value of the ΔBIC to convey support of a model against an alternative (i.e., Bayes factor > 1; Table 1).

#### Quantification of oscillations

We implemented a Matching Pursuit (MP) algorithm to quantify the strength of oscillations in the neural firing rate and local field potential (LFP) (Mallat and Zhang, 1993; Chandran et al., 2016). MP is a greedy algorithm designed to represent a finite signal, *s*(*t*), as a sum of Gabor functions (atoms) from a library that covers the position *τ* and width *σ* of the Gaussian envelope as well as the angular frequency *ξ* of the carrier sinusoids as follows:

$$g_{\gamma }(t)=\frac{1}{\sqrt{\sigma }}e^{-\pi {\left(\frac{t-\tau }{\sigma }\right)}^{2}}e^{i\xi t}$$

where the subscript, *γ*, identifies the atom, γ = {τ, σ, ξ}. MP is well suited to brief epochs containing mixtures of transient and periodic features. We used the open source algorithm developed by the Epilepsy Research Laboratory at Johns Hopkins Medical Institutions and Supratim Ray (available from https://github.com/supratimray/MP). For the spike rates, *s*(*t*) is the average firing rate, across trials, for a neuron, as shown in the example neurons (e.g., using –0.3 ≤ *t* < 0.724 s relative to the event of interest). The rates are computed in 1 ms bins without additional smoothing. For LFP data, the input is the trial averaged voltage (1 kHz sampling rate). The output is power as a function of time and frequency, as shown in Figure 5*B*. We define the low-beta power as the mean Wigner-Ville power ( ${\bar{P}}^{12:20}$) in the frequency band 12-20 Hz in the 90 ms before or 40-130 ms after event onset, denoted ${\bar{P}}_{\text{pre}}^{12:20}$ and ${\bar{P}}_{\text{post}}^{12:20}$, respectively. We typically report the mean ${\bar{P}}^{12:20}$ across neurons (± SEM) and determine statistical significance by applying a Wilcoxon signed-rank test (a nonparametric equivalent of the paired *t* test), using ${\bar{P}}_{\text{pre}}^{12:20}$ and ${\bar{P}}_{\text{post}}^{12:20}$ for each neuron. For comparisons of unpaired ${\bar{P}}_{\text{post}}^{12:20}$, we use the Mann–Whitney *U* test.

**Figure 4.**
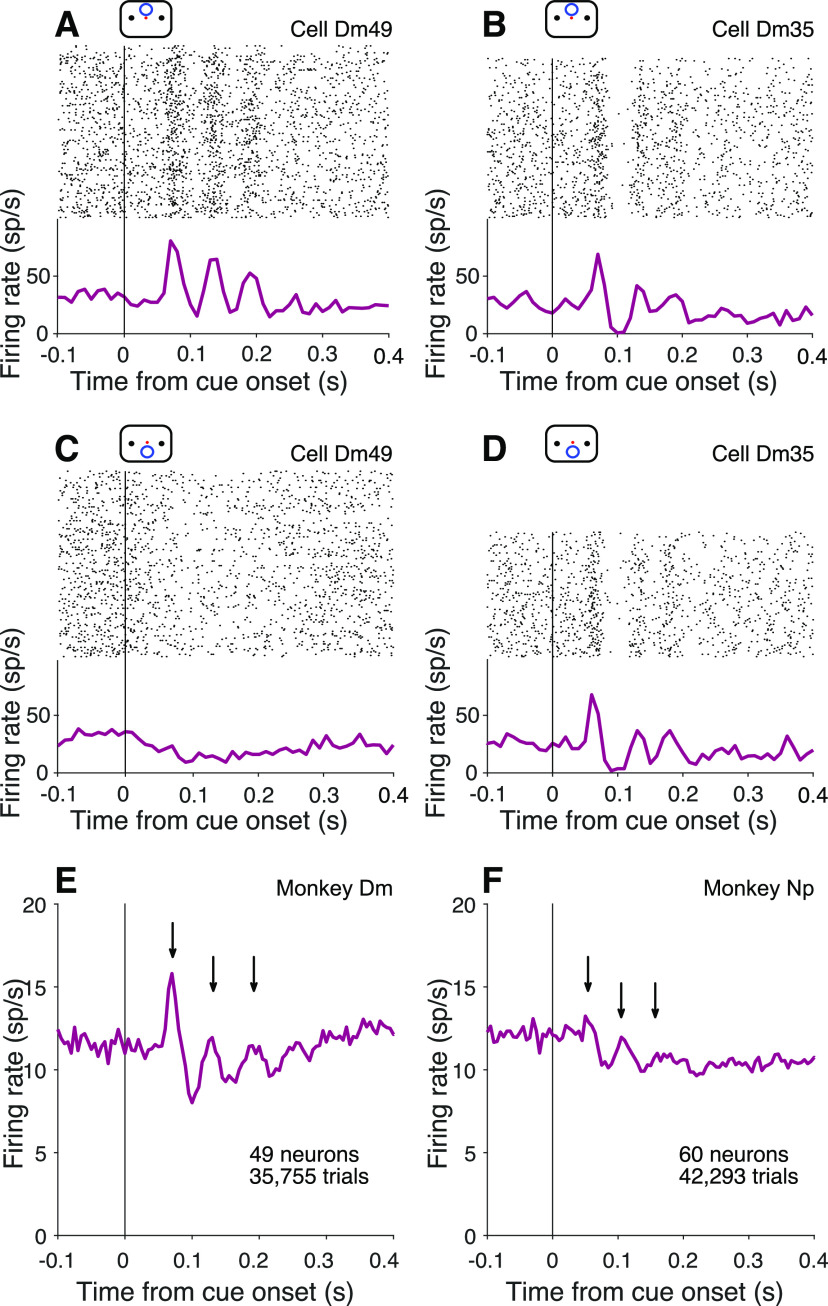
Oscillations at cue onset in the cued attention task. ***A***, ***B***, Activity of example neurons with the cue above fixation. Top, Raster plot of spike times relative to onset of the attention cue. Bottom, Peri-event histogram shows average firing rates across trials (bin width 10 ms). ***C***, ***D***, Cue aligned activity of the same example neurons for trials with the cue below fixation. ***E***, ***F***, Average activity across all neurons from Monkeys Dm (***E***) and Np (***F***), combining both cue locations (bin width 5 ms). Arrows indicate peaks after cue onset.

**Figure 5.**
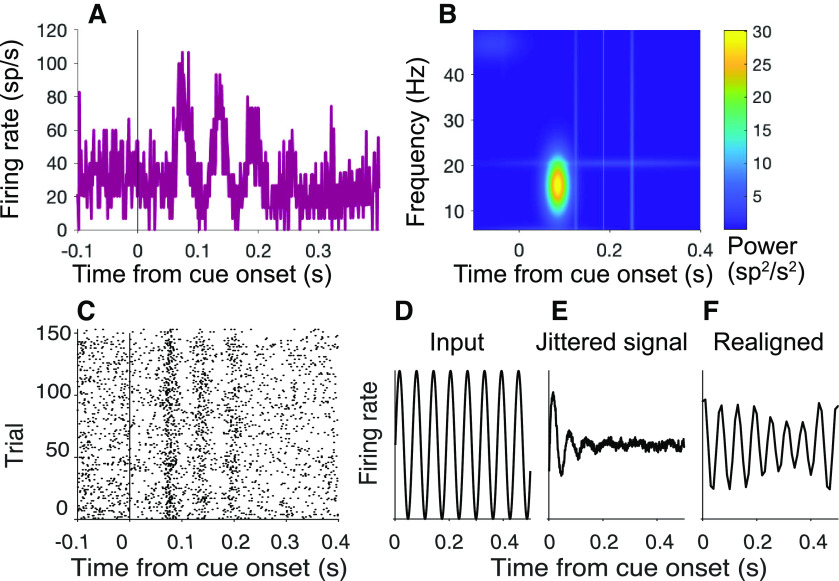
Detection of oscillations. ***A***, ***B***, Oscillations in spiking activity measured using the MP algorithm. The algorithm uses a greedy method to fit the waveform using a dictionary of Gabor functions of time (Eq. 6). ***A***, Input to MP algorithm. The average firing rate is rendered as a peristimulus time histogram (1 ms bin width) aligned to cue onset (neuron Dm49; same 150 trials shown in Fig. 4*A*). ***B***, Output of MP algorithm. Heat map represents power (color) by frequency and time from cue onset. ***C–F***, Realigning does not identify additional peaks. ***C***, Activity of example cell Dm49 at cue onset realigned using the affinewarp algorithm (Williams et al., 2020). ***D–F***, Affinewarp can recover temporally jittered oscillations in synthetic data. ***D***, Input oscillation. ***E***, Mean activity of 500 simulated trials using the firing rate function in ***D***, with added temporal jitter. For each trial, temporal noise is added at each time point before generating spikes, causing the oscillations to become misaligned in time and to disappear from the average. ***F***, Average firing rate of the activity of the trials shown in ***E*** after applying the affinewarp algorithm. The underlying oscillation is partially recovered.

The estimate of ${\bar{P}}^{12:20}$ can be impacted by the number of samples in the mean firing rate or LFP. For comparisons between conditions with unequal numbers of trials, we also evaluated the mean difference in ${\bar{P}}^{12:20}$ derived from random subsets of *N*_10_ trials from the two conditions, where *N*_10_ is ∼10% of the number of trials in the condition with the lesser number of trials. We calculated ${\bar{P}}^{12:20}$ from the spike rate averages in the two conditions and took the difference, $D_{\text{pow}}$. We repeated this procedure 1000 times and used the mean, ${\bar{D}}_{\text{pow}}$, as the estimate. We compared this test statistic to its distribution under the null hypothesis, by repeating the identical procedure on random subsets drawn from the union of the data from the two conditions, again using 1000 repetitions to achieve a sample of ${\bar{D}}_{\text{pow}}$ under the null hypothesis. We repeated this 100 times to estimate its distribution, and calculated the *p* values from the tail probabilities (two-tailed). This bootstrap procedure produces qualitatively similar results to those obtained from the Mann–Whitney *U* in almost all cases (e.g., comparison of ${\bar{P}}^{12:20}$ triggered by motion onset in the old datasets). The one exception is the comparison of ${\bar{P}}^{12:20}$ after motion onset on blocks of variable location and fixed location of the motion stimulus. This is why we qualify our interpretation of this finding.

For single-neuron analyses, the data from each neuron were divided into 50 trial blocks. For each block, we obtain ${\bar{P}}_{\text{pre}}^{12:20}$ and ${\bar{P}}_{\text{post}}^{12:20}$ and applied a Wilcoxon signed rank test to evaluate the null hypothesis of identical means.

#### Cell type analysis

Spike waveforms were preserved for 42 neurons from Monkey Np. Neurons were classified as putative excitatory or inhibitory based on the interval Δ between the peak and trough of the average spike waveforms. All but two were classified as either putative inhibitory (Δ ≤ 150 µs) or excitatory (Δ ≥ 350 µs) (Barthó et al., 2004; Ardid et al., 2015; Trainito et al., 2019).

#### Error analysis

To identify a relationship between the oscillations and task performance, we exploited the fact that some neurons show stronger oscillations for one cued location over the other. For the analysis in Figure 7, we calculated ${\bar{P}}_{\text{post}}^{12:20}$ separately for each neuron using the two cued motion stimulus locations, using both correct and error trials combined. Whichever location had the greater oscillation strength was deemed the “preferred” location. We calculated the ratio of ${\bar{P}}^{12:20}$ at preferred to nonpreferred locations. Using all trials, the geometric mean is >1, by definition. We then calculated the ratio separately for correct and error trials, retaining the original designation of preferred and nonpreferred location. We did not pursue this analysis using the LFPs because their amplitudes do not vary as a function of the location of the cued patch (*p *>* *0.05 for all neurons, permutation test, range 0.06–0.91, median 0.29).

#### Spike-field alignment

To assess the relationship between the oscillations in firing rate and LFP, we estimated the phase of the LFP associated with all spikes that occur in an epoch 40-130 ms after cue onset. The analysis was restricted to neuron-LFP recordings where the MP algorithm identified oscillations in the LFP (31 of 104 experiments; cued attention task), based on the criterion that at least one of the 10 strongest atoms (Gabor functions) overlaps the epoch and frequency band of interest (i.e., 12-20 Hz), based on its carrier. We used the inverse cosine of the carrier that overlaps the epoch and frequency band of interest to associate the time of each spike with a phase. Thus, spikes occurring near the peak or trough of the oscillation are assigned phases *ϕ_s_* ≈ 0 and *ϕ_s_* ≈ π, respectively. To produce the histogram of phase values in Figure 8*D*, we corrected for the nonuniform representation of cosine phase in the sampled epochs. We divided the number of occurrences of spike phases (*ϕ_s_*) in each 10° bin by the number of occurrences of the phase values contributed by the atoms. The latter is the distribution of all candidate phases within the sampled epochs. We evaluated the null hypothesis that *ϕ_s_* is uniformly distributed by comparing the cumulative distribution of *ϕ_s_* to the cumulative distribution of candidate phases (Kolmogorov–Smirnov test).

**Figure 6.**
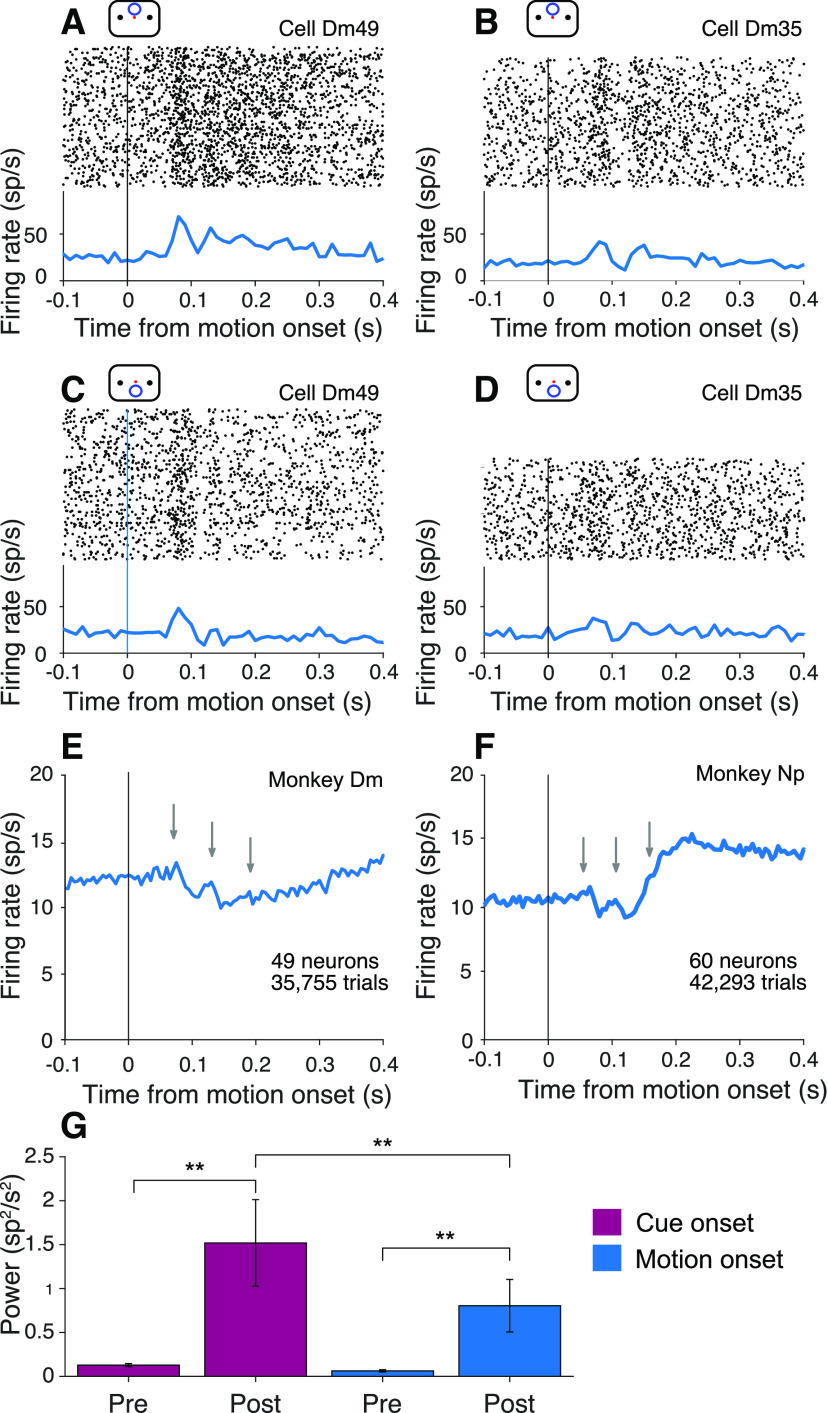
Oscillations at motion onset in the cued attention task. ***A****–****D***, Activity of the neurons shown in Figure 4*A-D*, aligned here to the onset of random dot motion. Attention was cued to the upper patch in ***A*** and ***B*** and to the lower patch in ***C*** and ***D***. ***E***, ***F***, Average activity across all neurons in both locations. Gray arrows indicate the positions of the peaks in activity in Figure 4*E*, *F*. ***G***, Average ${\bar{P}}_{\text{pre}}^{12:20}$ and ${\bar{P}}_{\text{post}}^{12:20}$, across all neurons from both monkeys, in 90 ms epochs before and after onset of the attention cue and random dot motion stimulus. *α = 0.05. **α = 0.01. Error bars indicate SEM.

**Figure 7.**
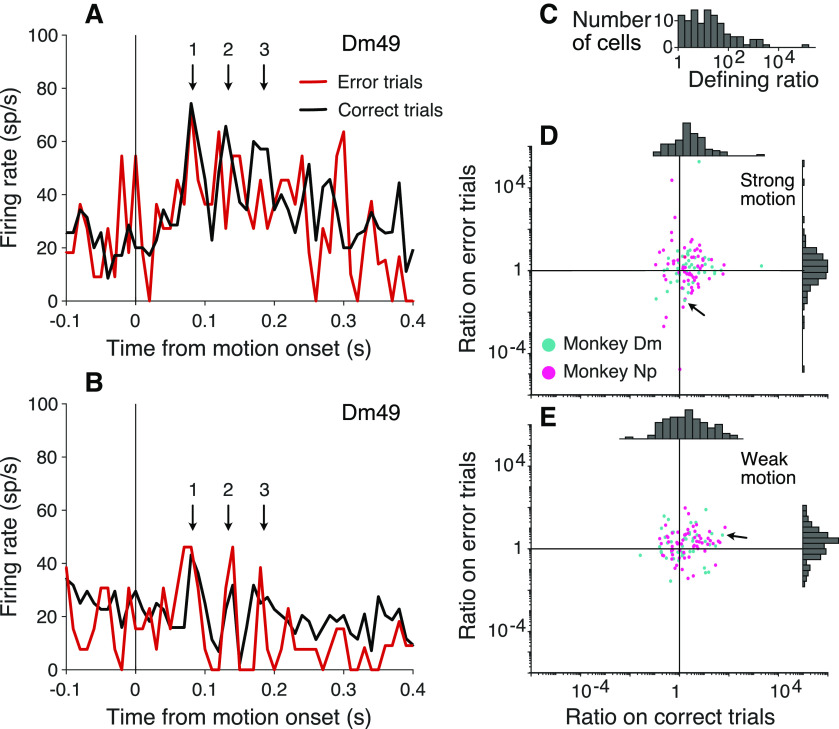
Oscillation amplitude tracks misrouting errors. The analysis exploits the observation that some neurons exhibit consistently stronger oscillations at the onset of motion when the upper or lower patch is cued. The example neuron (Dm49) shows stronger oscillations when the upper patch is cued. ***A***, Activity of Dm49 aligned to the onset of strong motion when the cued patch is above the FP. Colors represent correct (black) and error trials (red). The oscillation is weaker on error trials (e.g., cycles 2 and 3; arrows). ***B***, Same as in ***A***, when the cued patch is below the FP. The oscillation is stronger on error trials (all three cycles). ***C***, Histogram of the ratio of oscillation strengths at the preferred over nonpreferred locations, across all trials. By definition, ratio > 1. Note the logarithmic scale. ***D***, ***E***, Scatter plots represent the ratio of oscillation strengths at the preferred over nonpreferred locations for each neuron on correct choices versus errors. Arrows in both graphs identify Dm49. ***D***, Trials with strong motion, where errors are predominantly because of misrouting. On correct trials, the preferred location is more strongly oscillating (ratio > 1). On error trials, many cells exhibit weaker oscillations when the preferred location is cued (ratio < 1). ***E***, Trials with weak motion, where errors are predominantly perceptual. The ratios do not differ for correct choices versus errors.

## Results

Four rhesus monkeys (*Macaca mullata*) were trained to perform variations of the random dot motion task that required trial-by-trial changes in routing. In the cued attention task (Fig. 1*A*), two patches of random dot motion were presented on each trial, preceded by a cue that indicated which location the monkey must attend to. The monkey received a reward if it chose the direction of motion in the cued patch. In the variable location task (Fig. 1*B*), just one patch of motion was presented, but its location was unpredictable.

In the cued attention task, performance accuracy improved as a function of viewing duration at a rate consistent with temporal integration of evidence to a stopping bound, as shown previously (Kiani et al., 2008) (Fig. 2*B*). However, neither monkey was able to fully ignore the uncued patch, as evidenced by the shallower choice function on the trials in which the motion patches had opposite directions (Fig. 2*A*, open symbols). At the strongest motion strength (±51% coh), errors occurred on 11% of trials when the patches contained opposite directions, compared with 3% when the patches shared the same direction. Some of these errors can be explained by a failure to route information from the appropriate patch. The curves in Figure 2*A*, *D* are fits to a drift-diffusion model that takes into account both motion strength and viewing duration. Importantly, it allows for the possibility that the uncued patch of dots is not fully suppressed, and that on a random fraction of trials, *λ*, the monkey bases the decision on that patch (see Behavior; Table 1). In the variable location task, the monkeys made <2% errors when motion was strongest, nearly all of which were on trials with duration of <0.3 s (Fig. 2*D*). This performance is comparable to similar tasks in which the location of the motion stimulus was predictable (Gold and Shadlen, 2000; Fetsch et al., 2014). For the monkey that performed a free response version of the task, both reaction time and choice depended on the strength of motion (Fig. 2*E*).

### Neural recordings

The data comprise 173 neurons from LIP of four monkeys (Table 2). All neurons were screened for spatially selective persistent activity in ODR tasks. One of the saccadic choice targets (*T*_in_) was placed in the neural response field, typically in the contralateral visual field (see Materials and Methods). Such neurons are known to reflect the accumulation of evidence bearing on the decision to choose *T*_in_. As shown in Figure 3, the neural response begins to reflect the direction and strength of motion ∼180-200 ms after motion onset, and this holds whether the source of evidence is from the upper or lower location. In the cued attention task, the decision-related activity is also affected by the direction of motion of the uncued patch (Fig. 3*C*,*D*), consistent with the higher error rate on trials with motion in opposite directions.

It thus appears that by 180-200 ms after the onset of motion, some mechanism must establish functional connectivity between LIP neurons that represent the choice targets and the relevant direction-selective neurons with receptive fields that overlap the motion stimulus. In the cued attention task, this routing might occur following the attention cue. In the variable location task, only the onset of motion is informative. In what follows, we demonstrate a brief oscillation in the firing rates of LIP neurons. We first characterize the timing and strength of the oscillations in the two tasks. We then report additional observations that suggest the oscillations are associated with a mechanism that establishes the functional connectivity between sources of evidence and the circuits in LIP that use this evidence to establish the relative priority of the choice targets. In the Discussion, we consider how such oscillations might bear on neural mechanisms of routing.

### Oscillations in the cued attention task

Figure 4*A–D* shows prominent oscillations in the firing rate of two example neurons, aligned to onset of the attention cue. Importantly, the cue, like the random dot motion, was presented outside the neural response field (see Mapping tasks). One of the neurons (Dm49) exhibits 3 or 4 evenly spaced periods of increased activity (∼16.7 Hz) when the cue signaled that the relevant patch of motion would appear in the upper location. The other neuron (Dm35) exhibits a similar period but with a more pronounced decay in amplitude, independent of whether the cue appeared in the upper or lower location. These examples are among the most vivid in the dataset. In most cases, the oscillations are imperceptible in the trial rasters. The examples also highlight heterogeneous features, such as the rate of decay and spatial preference. What stands out as consistent is the timing, periodicity, and transient nature of the oscillations. These features are preserved in the firing rate averages across the population of neurons (Fig. 4*E*,*F*).

To further characterize the amplitude and frequency of these oscillations, we applied an MP algorithm (Mallat and Zhang, 1993; Chandran et al., 2016) to the the across-trial average spike rate functions for each neuron. MP is especially useful for brief periodic signals, as it measures power with high temporal resolution (see Materials and Methods). We report the average Wigner-Ville power, $\bar{P}$, in the 12-20 Hz range using 90 ms epochs preceding and following onset of the attention cue, denoted ${\bar{P}}_{\text{pre}}^{12:20}$ and ${\bar{P}}_{\text{post}}^{12:20}$ (–90 ≤ *t* < 0 and 40 ≤ *t* < 130 ms, respectively). We use the same nomenclature below, when aligning the response to other task events. The superscript identifies the range of frequencies contributing to the $\bar{P}$ statistic. The majority of neurons recorded in the cued attention task exhibit an increase in ${\bar{P}}^{12:20}$ after cue onset ( ${\bar{P}}_{\text{post}}^{12:20}$ > ${\bar{P}}_{\text{pre}}^{12:20}$, *p *<* *0.05, 65 of 109 neurons). Thirteen neurons show a significant difference in oscillation strength between locations, like that seen in example cell Dm49 (*p *<* *0.05, permutation test). The presence of oscillations is similar for putative excitatory and inhibitory neurons (ascertained from spike waveform analysis; see Cell type analysis). Across the population, the mean ${\bar{P}}_{\text{post}}^{12:20}$ was 1.52 ± 0.49 sp^2^s^–2^, an order of magnitude larger than ${\bar{P}}_{\text{pre}}^{12:20}$ (0.12 ± 0.03 sp^2^s^–2^; *p *<* *0.0001). In comparison, Wigner-Ville power in the 4-11 Hz band does not undergo change ( ${\bar{P}}_{\text{pre}}^{4:11}$ and ${\bar{P}}_{\text{post}}^{4:11}$ are 1.27 ± 0.29 and 1.32 ± 0.27 sp^2^s^–2^, respectively; *p *=* *0.35).

The oscillation in firing rate is triggered by the onset of the cue, and decays quickly thereafter. By 0.18 s after cue onset, ${\bar{P}}^{12:20}$ is only 0.067 ± 0.026 sp^2^s^–2^, which is comparable to ${\bar{P}}_{\text{pre}}^{12:20}$ (*p *=* *0.09). We wondered whether the oscillations are truly brief or are simply undetectable as a consequence of dephasing. To test this, we used a piecewise linear time warp designed to realign temporally jittered oscillations (Williams et al., 2020). While this algorithm successfully realigned jittered synthetic data, it did not identify any new peaks in the neural data (Fig. 5*C–F*). Nor does the oscillation strength depend on the duration of the cue. The difference in oscillation strength between trials in the shortest and longest quartiles is not significant (*p *=* *0.26). We therefore conclude that the oscillation is indeed short-lived and, in this case, caused by a task-relevant visual cue, outside the neural response field.

A weak oscillation in the firing rate is also present after motion onset. Figure 6*A–D* shows the activity of the same example neurons shown in Figure 4*A–D*, aligned to onset of the random dot displays. The oscillations are apparent in the rasters and average firing rates for both neurons. As shown in Figure 6*E*, *F*, they are also evident in the average firing rate across the population of neurons. They are weaker than the oscillations induced by the attention cue (*p *<* *0.0001; Fig. 6*G*), but they are statistically reliable: ${\bar{P}}_{\text{post}}^{12:20}$ is an order of magnitude larger than ${\bar{P}}_{\text{pre}}^{12:20}$ (0.80 ± 0.30 vs 0.06 ± 0.01 sp^2^s^–2^, *p *<* *0.0001). The weaker oscillation following motion onset is consistent with the hypothesis that these oscillations play a role in establishing functional connectivity. In the cued attention task, information about the location of the relevant motion patch was already supplied by the cue. We wondered why oscillations would be present at motion onset at all. One possibility is that the routing errors inferred from Equation 4 occur when the monkey forgets the cue in the interval preceding motion onset. If routing is reestablished when the two motion patches are displayed, it is possible that the uncued patch would supply the evidence. We therefore looked for a relationship between performance and oscillations at motion onset.

We compared oscillation strength on correct and error trials, when the upper and lower patches contained motion in opposite directions. We exploited the serendipitous observation, mentioned above, that some neurons exhibit a stronger oscillation at motion onset when either the upper or the lower motion patch was cued (e.g., compare Fig. 6*A*,*C*; *p *<* *0.01). The observation raises the possibility of associating some errors with misrouting. Recall that errors on trials with strong motion are often attributed to misrouting from the uncued patch (Fig. 2*A*). On the other hand, when motion strength is weak, errors are dominated by signal-to-noise considerations (i.e., perceptual errors). It is not possible to identify individual misrouted trials, but they ought to comprise a larger fraction of the errors on trials with stronger motion strengths.

Figure 7*A*, *B* shows the activity at motion onset of the example cell Dm49 for the two cued patch locations on correct and error trials, using only trials with strong motion in opposite directions. The neuron exhibits stronger oscillations when the upper patch was cued. Moreover, on these trials, the oscillations are more prominent on correct choices than on errors (Fig. 7*A*), especially the second and third cycles (arrows). However, when the lower patch was cued, the oscillations are stronger on the error trials (Fig. 7*B*). Both observations are consistent with the idea that errors on trials with strong motion may be attributed to misrouting. The oscillation strength depends on the source of the motion evidence, and the inclusion of misrouted trials shifts the average oscillation strength on error trials toward the oscillation strength on trials correctly routed from the opposite location. This pattern is evident across the population of neurons (Fig. 7*D*,*E*).

We introduced a ratio that captures this feature regardless of whether the stronger oscillation was associated with routing from the upper or lower patch. For each neuron, we used all trials to establish whether cueing the upper or lower patch led to the larger oscillation at motion onset, and we term this the neuron’s preferred location. The ratio of oscillation amplitudes from the two cued locations (preferred over non-preferred) is therefore greater than unity, by definition (Fig. 7*C*). Figure 7*D* focuses on trials with strong motion, grouping the data by correct versus error for trials with strong motion. On correct choices, the ratios of amplitudes on trials when the preferred versus nonpreferred locations were cued tend to retain their original form, lying mainly to the right of the vertical axis (ratio > 1). However, on the error trials, the distribution of ratios picks up more mass below the horizontal axis (ratio > 1) as if some ratios were flipped to their reciprocal. In other words, when an error occurs on a trial where the cued location is the neuron’s preferred location, the neuron exhibits oscillations with amplitude associated with its nonpreferred location. When the cued location is the neuron’s nonpreferred location, the neuron exhibits oscillations with amplitude associated with its preferred location. The pattern is consistent with the hypothesis that some errors arise through misrouting. The presence of these routing errors leads to a mixture of routing from both patches on these error trials, eroding the distinction between the strongly and weakly oscillating locations. The net effect is a difference in the distributions projected on the two axes (Kolmogorov–Smirnov test, *p *<* *0.0005). On low coherence trials (Fig. 7*E*), the majority of error trials are perceptual. Routing errors are still present, but they are overwhelmed by the more prevalent perceptual errors. Routing errors may even occur on correct trials, if there were also a perceptual error. Accordingly, on these low coherence trials, there is not a statistically reliable difference between ratios on correct and error trials (*p *=* *0.56). Not all errors are explained by misrouting. Were that the case, there should be few points in the upper right quadrant of the scatter plot in Figure 7*D*. This is consistent with the idea that some high coherence errors occur despite successful routing, but are instead attributed incomplete suppression of the irrelevant patch (Table 1).

**Figure 8.**
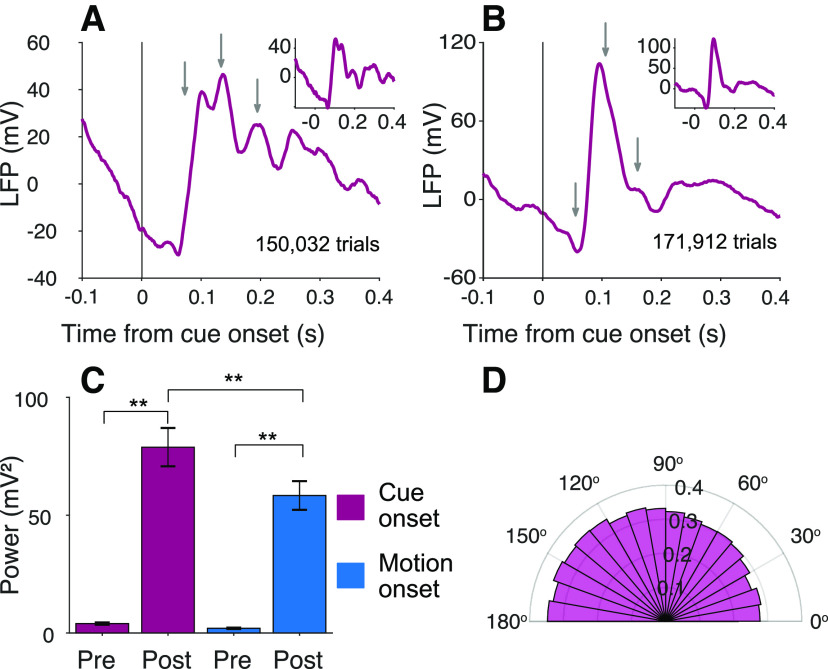
Oscillations are present in the LFP. ***A***, ***B***, Average baseline-corrected LFP from monkeys Dm and Np, respectively, aligned to onset of the attention cue in the cued attention task. Gray arrows indicate the peaks in firing rate activity shown in Figure 4*E*, *F*. Insets, The same signal for channels where no neurons were identified. ***C***, Average ${\bar{P}}_{\text{pre}}^{12:20}$ and ${\bar{P}}_{\text{post}}^{12:20}$, across all sites and both monkeys, measured in 90 ms epochs preceding and following onset of the attention cue and random dot motion. Same conventions as in Figure 6. ***D***, Phase alignment of spikes with LFP. Histogram represents the number of spikes in 30° bins of phase. The phases refer to the cosine carrier of the best Gabor. There is a tendency for spikes to occur more frequently near the trough of the LFP (see Spike-field alignment).

We also detected oscillations in the LFP recordings made from the same electrode used for the neural recordings. The LFPs revealed oscillations similar to those detected in the spiking activity. For example, Figure 8*A* shows the average LFP for Monkey Dm, aligned to the onset of the attention cue. The gray arrows are copies of the black arrows in Figure 4, which show the peaks in the firing rate oscillations from the same experiments. The oscillations in the LFP recordings from Monkey Np are less pronounced, but some deflection is evident at the time of the peaks in spike rate, shown by the gray arrows in Figure 8*B*. For both monkeys, ${\bar{P}}_{\text{post}}^{12:20}$ is greater than ${\bar{P}}_{\text{pre}}^{12:20}$ for both cue and motion onset (*p *<* *0.0001); and like the firing rate oscillations, ${\bar{P}}^{12:20}$ at motion onset is weaker than at cue onset (*p *<* *0.0001, Fig. 8*C*).

The oscillations in the LFP and firing rates appear to be manifestations of a common underlying mechanism. In addition to the similarity in their timing and frequency, there is a tendency for spikes to occur in the trough of the LFP oscillation (*p *<* *0.0005; Fig. 8*D*). The frequency histogram of spike phases is obtained by extracting the dominant Gabor atom from the MP analysis of the LFP. The spike phases are defined as the inverse cosine of the atom’s carrier sinusoid at the time of the spike, such that zero and *π* are the peak and nadir, respectively (see Spike-field alignment). The observation is unsurprising given the similarity of the signals, but it is not an artifact of recording the LFP and action potentials from the same electrode. Similar oscillations in the LFP are present at electrodes that pick up few (or zero) spikes (Fig. 8, insets).

### Oscillations in the variable location task

**Figure 9.**
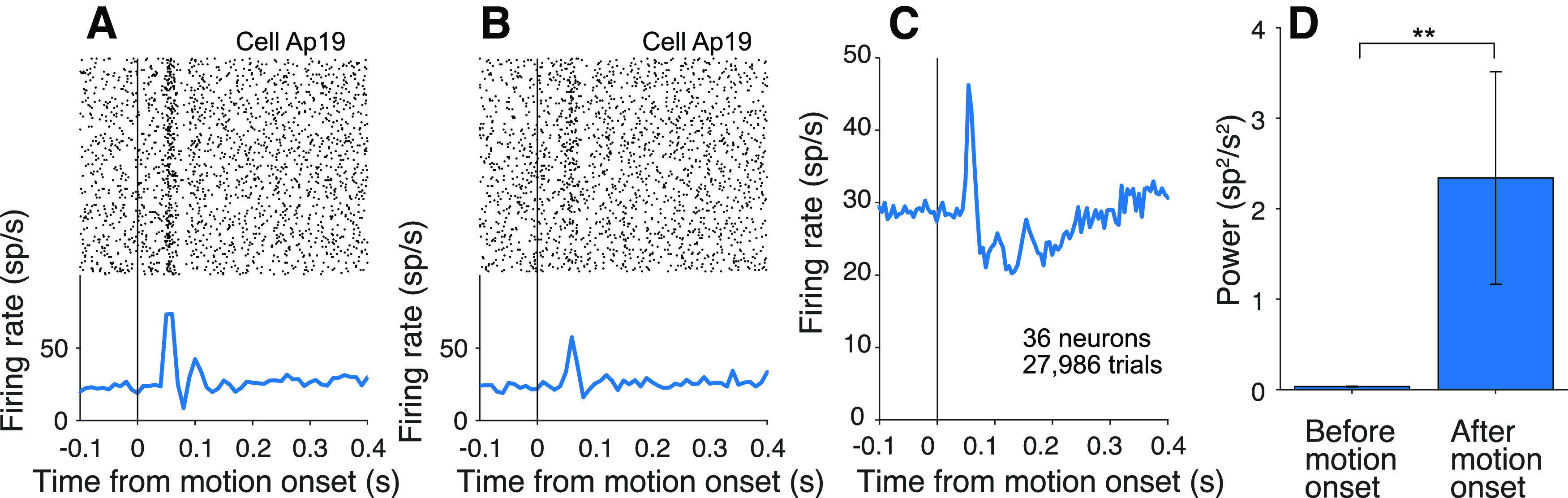
Oscillations in the variable location task. ***A***, Activity of an example neuron with stimulus presented above fixation. Top, Raster plot of spike times relative to onset of random dot motion. Bottom, Average firing rates across trials (computed in 10 ms bins). ***B***, Same neuron on trials when the motion stimulus appeared below fixation. ***C***, Average firing rate, across all neurons from Monkeys Ap and Dz. ***D***, Average ${\bar{P}}_{\text{pre}}^{12:20}$ and ${\bar{P}}_{\text{post}}^{12:20}$, across all neurons, measured in 90 ms epochs preceding and following onset of random dot motion. Same conventions as Figure 6*G*.

In the variable location task, it is the appearance of the random dot motion itself that resolves the uncertainty about the source of evidence bearing on the decision. As in the cued attention task, the routing must be established between direction selective neurons in the visual cortex that represent the motion and the LIP neurons that represent one of the choice targets. Here, however, connectivity must be established between the onset latency of visual cortical neurons and the beginning of evidence accumulation, ∼40-180 ms from motion onset. The example neuron shown in Figure 9*A*, *B* exhibits oscillations in the firing rates similar to those in the cued attention task. They are also evident in the pooled firing rates across 64 neurons from the three monkeys (Fig. 9*C*). The average ${\bar{P}}_{\text{post}}^{12:20}$ is two orders of magnitude larger than ${\bar{P}}_{\text{pre}}^{12:20}$ (2.3 ± 1.2 and 0.034 ± 0.005 sp^2^s^–2^, respectively; *p *<* *0.0001 with or without Monkey Dm).

The task comprises alternating blocks of fixed and variable stimulus locations. In the former case, it may not be necessary to establish the appropriate functional connectivity on every trial. We therefore predicted that signals associated with routing might be diminished in these blocks. Indeed, ${\bar{P}}_{\text{post}}^{12:20}$ is slightly reduced in blocks where the motion stimulus location was fixed (*p *<* *0.01, permutation test, with or without Monkey Dm). While significant, there are aspects of the design that might weaken this comparison. In particular, the monkey had learned to expect the motion stimulus to appear in various locations, and may not have adapted fully to the blocked design. We therefore augmented this analysis with a reanalysis of two older datasets, which are better suited to test our hypothesis.

**Figure 10.**
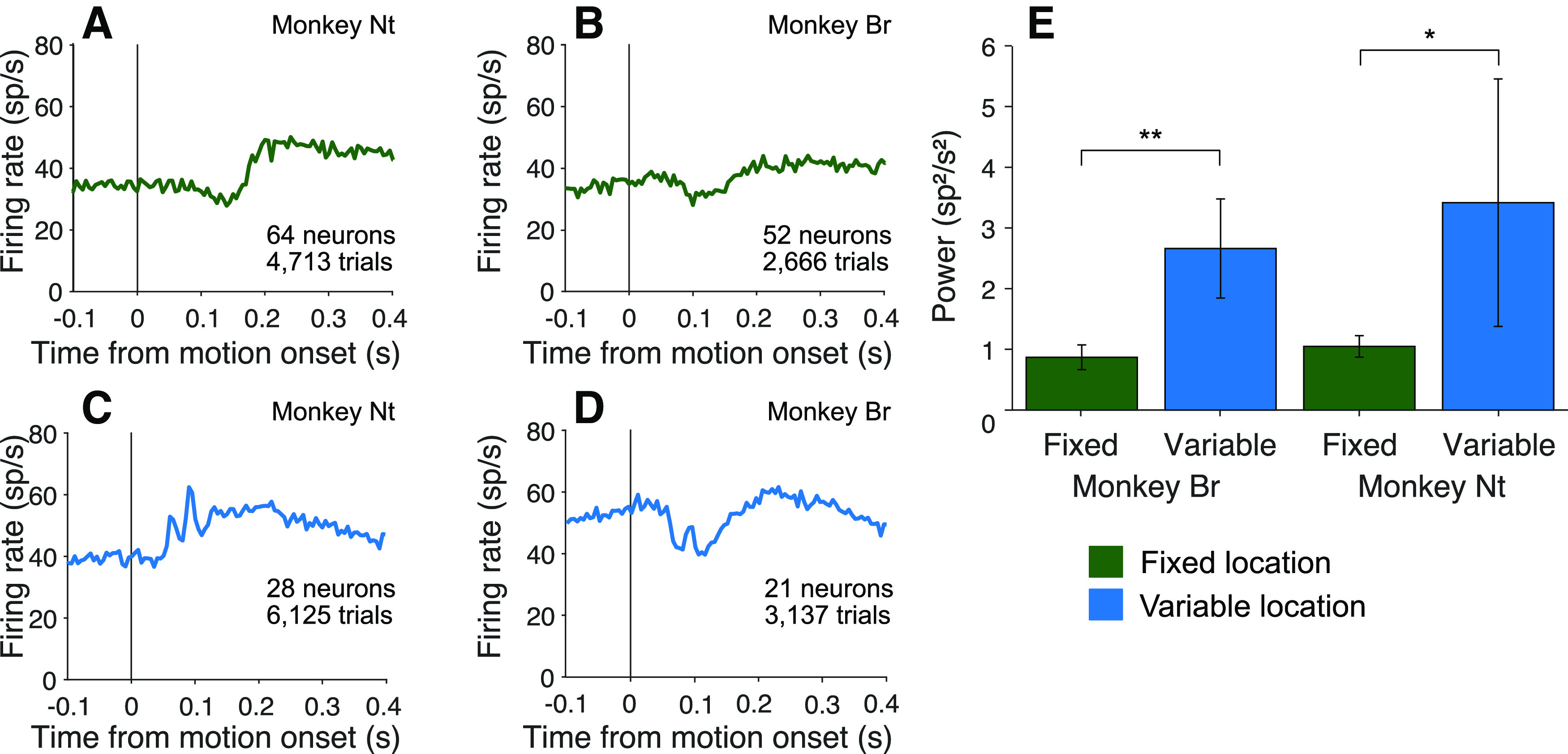
Oscillations emerge after encountering conditions necessitating flexible routing. Two monkeys were first trained and tested with random dot motion stimuli, presented at one central viewing location (fixed location). They were subsequently trained and tested with stimuli presented at a variety of locations (variable location). ***A***, ***B***, Average firing rate aligned to motion onset in the fixed location experiments. ***C***, ***D***, Average firing aligned to motion onset in the variable location experiments. ***E***, Comparison of Average ${\bar{P}}_{\text{post}}^{12:20}$ in the two conditions, by monkey. Same conventions as in previous bar graphs.

Two monkeys reported in Roitman and Shadlen (2002) were trained and studied with random dot motion viewed at the center of the visual field. One year later, the same monkeys were retrained and studied on a variable location task (included in Shushruth et al., 2018). As shown in Figure 10, we did not detect oscillations in the recordings from either monkey in the earlier fixed location study, whereas they are clearly present in the data from the same monkeys — and same LIP — in the variable location design (Table 3). While the study was not designed with this longitudinal comparison in mind, it provides support for the hypothesis that the transient oscillations are associated with neural mechanisms responsible for flexible routing. It also rebuts the assertion that the oscillations are triggered by any task-relevant visual stimulus. The oscillations appear to be associated with task events that resolve uncertainty about the source of information.

**Table 2:** Number of neurons recorded from each monkey in the tasks^*a*^

	Np	Dm	Ap	Dz	Nt^*^	Br^*^
Cued attention	60**	49**	—	—	—	—
Variable location	—	28	23	—	—	—
Variable location (FR)	—	—	—	13	28	21
Fixed location (FR)	—	—	—	—	64	52

*^a^*FR, Free response task.

* Previously published data from Roitman and Shadlen (2002) and Shushruth et al. (2018).

** Recorded with 16-channel probe.

**Table 3:** Oscillation power in fixed and variable stimulus location tasks^*a*^

	Fixed location (FR)			Variable location (FR)		
	${\bar{P}}_{\text{pre}}^{12:20}$	${\bar{P}}_{\text{post}}^{12:20}$	*p*	${\bar{P}}_{\text{pre}}^{12:20}$	${\bar{P}}_{\text{post}}^{12:20}$	*P*
Nt	0.8 ± 0.2	1.0 ± 0.2	0.06	0.3 ±0.1	3.4 ± 2.0	0.04
Br	0.8 ± 0.1	0.9 ± 0.2	0.61	0.2 ± 0.1	2.2 ± 1.0	0.0007
Combined	0.88 ± 0.12	0.97 ± 0.13	0.11	0.40 ± 0.10	2.6 ± 0.9	0.00001

*^a^*Units are sp^2^s^–2^. FR, Free response task.

**Figure 11.**
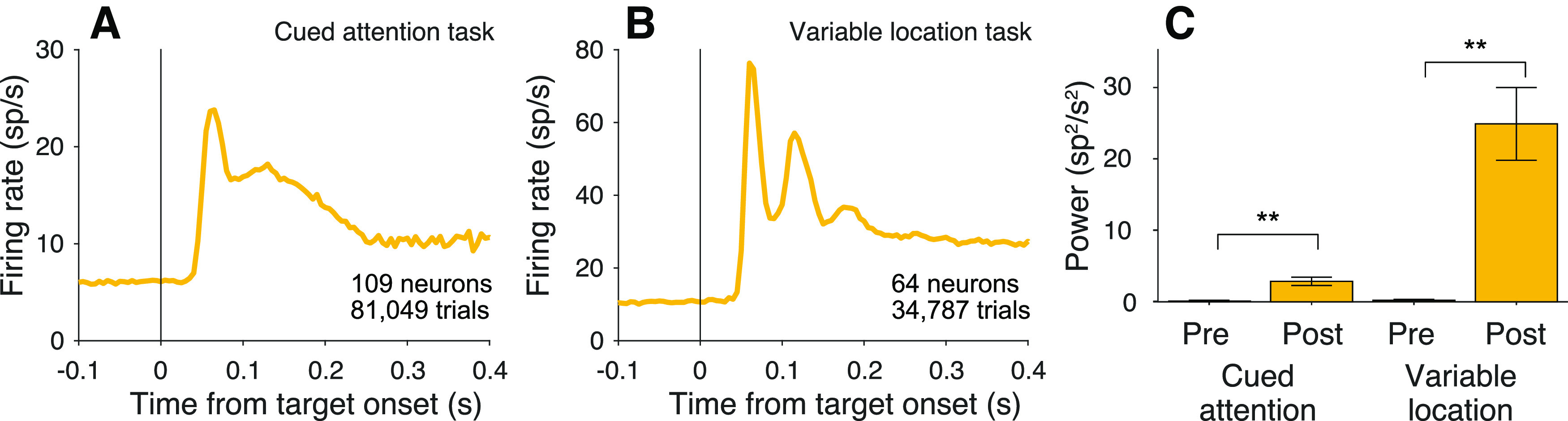
Oscillations are present at target onset. ***A***, Average firing rate aligned to onset of the choice targets for both monkeys in the cued attention task. ***B***, Average firing rates aligned to onset of the choice targets for all three monkeys in the variable location task. ***C***, Average ${\bar{P}}_{\text{pre}}^{12:20}$ and ${\bar{P}}_{\text{post}}^{12:20}$, across neurons, measured in 90 ms epochs preceding and following onset of targets. Same conventions as in previous bar graphs.

**Figure 12.**
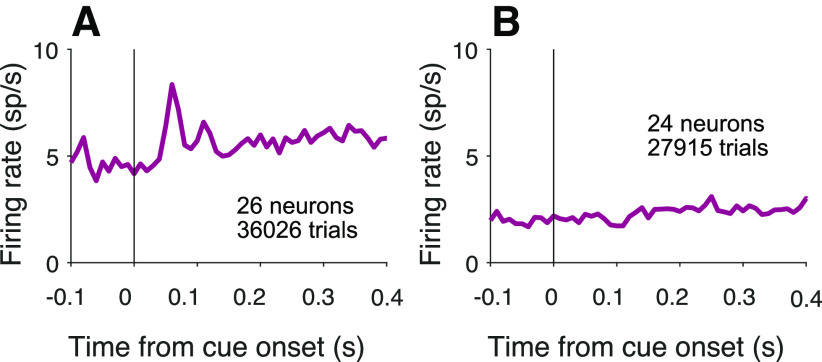
Oscillations are present only when a task-relevant object is in the response field. Here we analyze neurons in the cued attention task that were excluded from previous analyses because neither choice target was in the response field. Responses are aligned to onset of the cue, and the averages are combined data from Monkeys Dm and Np. ***A***, Average firing rate of neurons with the cue overlapping the response field. ${\bar{P}}_{\text{post}}^{12:20}$ is greater than ${\bar{P}}_{\text{pre}}^{12:20}$ (*p *<* *0.01). ***B***, Average firing rate of neurons with no visual stimuli in the response field. There is no detectable increase in oscillation strength from ${\bar{P}}_{\text{pre}}^{12:20}$ to ${\bar{P}}_{\text{post}}^{12:20}$ (*p *=* *0.37).

Of course, routing requires specification of both the source and destination of information. We therefore looked for oscillations following the onset of the choice targets. Recall that this event precedes the attention cue in the cued attention task, and it precedes the motion onset in the variable location task. As shown in Figure 11, oscillations are present in both tasks following onset of the choice targets (*p *<* *0.0001), one of which is in the neural response field. The oscillations can also arise when both targets appear outside the neural response field, but are relevant to the routing of other information in the response field. For example, this occurs in the second of the reanalyzed datasets, where in some blocks the motion stimulus is displayed in the neural response field and the choice targets, therefore, are not (0.21 ± 0.07 and 0.54 ± 0.11 ${\text{sp}}^{2}{\text{s}}^{-2}$ for ${\bar{P}}_{\text{pre}}^{12:20}$ and ${\bar{P}}_{\text{post}}^{12:20}$, respectively; *p *<* *0.001). This observation, like the attention cue in Figure 4, is another example of an oscillation caused by the onset of stimuli outside the neural response field, but relevant to neurons that represent the predicted retinotopic location of the motion evidence. In the cued attention task, oscillations are present at cue onset when a target is located in the response field (Fig. 4) or when the target is outside the response field, but the cue overlaps it (Fig. 12*A*). However, oscillations are not detectable when all task-relevant stimuli are located outside the response field (Fig. 12*B*). We interpret this as further support that the oscillations are not associated with all visual objects but those that are plausibly linked with other objects in some task-relevant way.

## Discussion

Unlike innate sensory-response programs, such as escape or courtship, evolution did not imbue the brain with circuits devoted to the vast repertoire of decisions one encounters in life, including the motion tasks studied here. Whereas the processing of motion and the organization of orienting eye movements rely on dedicated sensory and motor circuits, the neural circuits responsible for planning possible eye movements cannot exploit dedicated connections to the neurons that represent all the possible sources of evidence bearing on such plans. The flexibility to learn which sources are relevant and to route them in the moment are hallmarks of higher brain function.

We studied an example of flexible routing by introducing uncertainty about the source of visual evidence bearing on a decision about motion direction. In one task, the location of a single patch of random dots was varied randomly across trials. In the other, an attention cue indicated which of two motion patches should inform the decision. In both tasks, the decision was communicated by a saccadic eye movement to one of two choice targets. An advantage of this highly studied perceptual decision is the accompanying quantitative framework that unites choice accuracy, sensitivity, decision time, change of mind, and confidence (Shadlen and Kiani, 2013). We exploited this framework to show that a portion of the errors on the cued attention task are explained by some form of misrouting. Both monkeys fail to suppress all information from the uncued patch, and Monkey Dm appears to attend to the wrong patch altogether on 3%-6% of trials, accounting for at least half of the errors at the strongest motion strength. The observations are consistent with a large body of work in cognitive science that frames attention in terms of the control of information flow (Posner, 1988; Driver, 2001; Posner et al., 2004; Buschman and Miller, 2009; Buschman and Kastner, 2015; Panichello and Buschman, 2021). In the present study, this control problem must be resolved by the time neurons in association cortex begin to integrate the sensory evidence toward a decision. The destination of the routed information is specified when the choice targets appear, but the source is uncertain until the onset of motion in the variable location task and the onset of the attention cue in the cued attention task.

We discovered a neural correlate of this routing event in the LIP. We focused our recordings on neurons with two properties: (1) a response field that overlaps one of the choice targets and (2) spatially selective persistent firing rates during an ODR task. Such neurons are known to represent the accumulation of the noisy evidence used by the monkey to inform the saccadic choice, and we replicated this phenomenon (Fig. 3). We observed that many such neurons also exhibit a brief oscillation in firing rate that is time-locked to the moment when information about the source of evidence becomes available. The oscillation manifests as a transient excess of spikes that repeats one or more times at intervals of 61 ± 2 ms (∼16 Hz), and it appears to be coupled to the LFP (Fig. 8). Striking examples like those in Figures 4, 6, and 9 are rare, but the majority of cells showed an increase in oscillatory activity in this range. These oscillations are undetectable in reanalyzed data from Roitman and Shadlen (2002), in which the motion stimulus was presented at the same location on all trials. The oscillations appear in the same monkeys (and same recording sites) after they were trained to base their decisions on stimuli that could appear in different locations (Fig. 10). This serendipitous observation supports the hypothesis that the phenomenon is associated with flexible routing of information from neurons that represent the stimulus motion to neurons in LIP that represent the decision. The connection path is almost certainly polysynaptic.

One might expect that a routing signal would appear only once per trial, when the information needed for routing is first received. However, in the cued attention task, the oscillations return, albeit weakly, after motion onset. This may be because, on some trials, routing is not maintained between cue and motion onset. This might explain why oscillations associated with motion onset are weaker, on average. More importantly, when routing is restored at motion onset, it may be incorrectly assigned to the wrong motion patch, leading to errors when the patches contain opposite directions. Based on analyses of behavior (Fig. 2), such “misrouting errors” should constitute a small fraction of the errors on trials with weak motion, whereas they should constitute the majority of the errors on trials with strong motion. This pattern is supported by the analysis in Figure 7.

Previous studies have identified transient oscillations, also in the low β range, that correlate with performance on perceptual tasks (Koelewijn et al., 2008; Haegens et al., 2011; Siegel et al., 2011). For example, Donner et al. (2007) describe such oscillations originating from posterior parietal cortex following onset of a random dot motion stimulus. They reported that power was greater when a stimulus was correctly categorized as motion or noise (hits and correct rejects) than on misses and false alarms. Relatedly, Fiebelkorn et al. (2013) reported periodicity in detection accuracy during visual detection tasks. The periodicity was synchronized with cycles of the theta rhythm measured in the LFP recorded from area LIP, and the poor-detection phases were associated with increased power in an associated 10-18 Hz frequency band (Fiebelkorn et al., 2018, 2019), which the authors interpret as a sign of attentional shifts away from the task. These shifts in attention might involve the same routing processes as allocation of attention in our task. In our task, errors were not associated with a failure to route, but rather, a failure to route the appropriate information. Therefore, there is little overall decrease in oscillation strength on error trials. However, as just mentioned, on trials with strong motion, we found a signature of misrouting in neurons that exhibited location-specific oscillations (Fig. 7).

Why would oscillations be associated with routing? One possibility is that they serve to synchronize spikes and thus increase their influence on downstream circuits (König et al., 1995; Singer and Gray, 1995; Fries, 2005, 2015; Buschman and Miller, 2009; Gregoriou et al., 2009; Akam and Kullmann, 2010). If so, they ought to be present during the epoch in which signals in upstream motion areas are affecting the LIP response. In our data, however, they are present only transiently, in the epoch preceding the transfer of information (cf. Panichello and Buschman, 2021). We therefore infer that they are associated with the mechanism that establishes the connection rather than facilitating the flow of information directly. Of course, the oscillations themselves do not form the connections, but they may provide a clue to the underlying mechanism. Among the many challenges posed by routing is the need to identify the appropriate neurons at the source and destination. We suspect that the oscillatory signal is linked to this identification function.

It has been shown that field potentials (e.g., eCoG) are associated with calcium plateau potentials in apical dendrites of layer 5 pyramidal neurons (Suzuki and Larkum, 2017), and these same potentials are capable of inducing plastic changes at relevant time scales (e.g., behavioral time scale plasticity) (Magee and Grienberger, 2020). Such plateau potentials and their biochemical sequelae might allow long range projections — especially feedback — to identify their targets, or for the targets of the projections to establish a state of receptivity to a signal that is broadcast widely (Quinn et al., 2021; So and Shadlen, 2022). Such a mechanism might allow feedback projections to pick out the causes of the activity that is feeding back. This might serve many functions, including learning to use those inputs again under the right conditions, or to bind in some way the cause of an event with its consequences. Oscillatory activity might be a signature of these inputs (Zhang and Bruno, 2019). In this sense, we are in agreement with a longstanding view that brain oscillatory activity might signify operations that firing rates alone do not divulge (Freeman et al., 1983; Singer and Gray, 1995; Fries et al., 2001; Crick and Koch, 2003).
